# Body Appreciation Scale (BAS-2): measurement invariance across genders and item response theory examination

**DOI:** 10.1186/s40359-021-00609-3

**Published:** 2021-07-30

**Authors:** Daniel Zarate, Joshua Marmara, Camilla Potoczny, Warwick Hosking, Vasileios Stavropoulos

**Affiliations:** 1 Victoria University, Melbourne, Australia; 2 Institute for Health and Sport, Victoria University, Melbourne, Australia; 3 Department of Psychology, University of Athens, Athens, Greece

**Keywords:** Body appreciation, Measurement invariance, Item response theory, Psychometric properties, Positive psychology, Gender

## Abstract

**Background:**

The present study considers a measure of positive body image, the Body Appreciation Scale-2, which assesses acceptance and/or favourable opinions towards the body (BAS-2). Potential variations of the psychometric properties of the scale across males and females, as well as across its different items invite for further investigation. The present study contributes to this area of knowledge via the employment of gender Measurement Invariance (MI) and Item Response Theory (IRT) analyses.

**Methods:**

A group of 386 adults from Australia, Canada, New Zealand, Ireland, the United Kingdom, and the United States of America (USA) were assessed online (N = 394, 54.8% men, 43.1% women, *M*_age_ = 27.48; SD = 5.57).

**Results:**

MI analyses observed invariance across males and females at the configural level, and non-invariance at the metric level. Further, the graded response model employed to observe IRT properties indicated that all items demonstrated, although variable, strong discrimination capacity.

**Conclusions:**

The items showed increased reliability for latent levels of ∓ 2 SD from the mean level of Body Appreciation (BA). Gender comparisons based on BAS-2 should be cautiously interpreted for selected items, due to demonstrating different metric scales and same scores indicating different severity. The BAS-2 may also not perform well for clinically low and high BA levels. Thus, it should optimally be accompanied by clinical interviews for formal assessment in such cases.

## Introduction

Body image is a multidimensional construct that represents one’s cognitions, behaviours, perceptions, and affective responses towards their body [10]. Contemporary literature has predominantly focused on negative body image and its relationship with poor mental health [3, 11]. Some studies appear to focus on a uni-dimensional component of body image by emphasizing a negative connotation, primarily related to mental health treatment seekers [39]. Such conceptual biases have been challenged by literature suggesting emphasis on the whole spectrum of body image variations, ranging from negative to positive [1, 8, 9, 19]. In this context, one’s body appreciation (BA) is linked with one’s positive body image. BA is depicted as “accepting and holding favourable opinions towards the body, while rejecting mainstream ideals of stereotypical human beauty” [41]. To measure BA, Avalos and colleagues [2] pioneered the body appreciation scale (BAS). The use of the BAS has demonstrated positive ties between BA and one’s psychological well-being (e.g., self-esteem, optimism, positive affect [2, 34], and negative links with body surveillance, body shame, and body dissatisfaction. These findings underpinned the introduction of an upgraded tool––the Body Appreciation Scale 2 (BAS-2, [41]) to eliminate sex-specific and body dissatisfaction-based language. Interestingly, this 10-item scale was originally devised to measure BA exclusively among women and was later modified to include men [41]. Thus, one could assume that the scale may operate differently between male and female respondents. This would mean that any BAS-based comparisons between the two genders could be confounded by the psychometric properties of the scale. This possibility highlights the importance of establishing the psychometric properties of the BAS-2 across traditional binary forms of gender (men and women). To address this aim, one would need to utilise Measurement Invariance (MI [27]).

### Measurement Invariance (MI)

Establishing MI across observed groups, such as genders, is of paramount importance to claim significance over inferred comparative observations [32]. MI can be considered as a test of heterogeneity that evaluates whether the measurement properties of a construct remain stable across groups, thus securing meaningful comparisons [33]. A comprehensive method to evaluate whether MI exists across groups is Multigroup Confirmatory Factor Analysis (MCFA, [4]). Rooted in Classical Test Theory (CTT), MCFA assumes that (a) observed scores are a result of adding true scores and error terms, that (b) true scores are the ideal value of a construct in an individual, and that (c) standard error of measurement applies to all scores in a particular population [14, 15]. In addition, this method involves evaluating whether significant differences in variance across groups exist at different/successive levels of the construct. These entail configural (i.e., factorial structure), metric (i.e., factor loadings), scalar (i.e., intercepts or thresholds) and strict (i.e., residuals) MI. In this case, confirming configural invariance would imply that the number of factors and pattern of item-factor loadings within the BAS-2 are similar for men and women. Similarly, achieving support for BAS-2 metric invariance would suggest that the item-factor loading relationship is being measured with the same metric scale for both groups. Last, achieving support for BAS-2 scalar invariance would suggest that item intercept values are equal across groups. Thus, males and females would be expected to rate each item similarly when experiencing the same level of BA. It is noted that testing for equality of error/residual variance across the groups as an additional layer of invariance is often disregarded. Due to residuals being expected to be random, testing their intergroup equality would represent excessively stringent criteria, and thus likely unnecessary and un-informative models [4].

Indeed, Avalos and colleagues’ [2] invitation for further investigation of the BAS/BAS-2 [41] equivalence of psychometric properties across the two genders has been evaluated via USA [40], Spanish [36], Polish [28], French [20], Danish, Portuguese, Swedish [22] and Chinese samples [35]. These studies concluded that gender MI was consistently achieved at the configural and metric levels, and usually (although not always) achieved at the scalar level (with Chinese and Danish samples observing non-invariance; [22, 35]). However, the applied criteria oscillated between a ‘more relaxed approach’ guided by difference between models in CFI and RMSEA values [13] and a ‘more stringent approach’ guided by difference in *χ*^2^ values [4]. Evaluating MI with a ‘more relaxed approach’ resulted in support for invariance (at all three levels) in the Polish sample and support for partial scalar invariance in the Portuguese, Danish and Swedish samples [22, 28]. Evaluating MI with a ‘more stringent approach’ resulted in support for invariance (at all three levels) in the Chinese, French and Spanish samples, and support for partial scalar invariance for the USA sample, after freeing items 1 and 9 to vary across gender groups (with original BAS comprising 13 items; [20, 35, 36, 40]). Nonetheless, correlated residuals were required in the French (6–10, 6–7, and 1–5 items) and Spanish (1–5, 2–9 and 8–10 items) samples to achieve acceptable fit indices [20, 35, 36]. Taken together, BAS-2 items may not only operate differently across genders, but also across different national samples. Given the importance of the construct, as well as its wide use in both clinical and community populations, further thorough examination of the BAS-2 psychometric properties is imperative.

### Item Response Theory (IRT)

Item Response Theory (IRT) projects as a superior way of assessing the psychometric properties of a scale at the item level. IRT is assumed to outperform CTT’s psychometric estimation, such as the BAS/BAS-2 MI analyses previously implemented, in a twofold manner [11]. First, while CTT explains relationships between underlying psychological attributes (i.e., latent trait) and items, IRT aims to explain how both the latent trait and item properties are related to individual responses to an item [14]. Second, unlike CTT, IRT assumes a non-linear standard error of measurement (SEM), that differs across levels of the latent trait in the same population [15]. Considering that IRT employs a framework for quantifying SEM as a function of item parameter (i.e., difficulty) and participant’s latent trait (*θ*), different/conditional reliability indices can be observed at different levels of the latent trait [21]. In other words, observed reliability scores are conditional on different participant ability (*θ*) and SEM [21]. In addition, IRT can generate reliability indices and standard errors for each item rather than just an overall reliability index. Thus, while CFA (CTT) aims to explain relationships between BAS-2 items and BA (as the latent construct), IRT models evaluate distinct relationships between items and participants’ responses to those items, whilst taking into consideration participants’ latent BA levels [4].

The item-participant relationship is represented by the probability that participants with a certain level of the latent trait (in this case BA) will endorse a particular item (for a detailed account of BAS-2 items see Table 1). This is graphically represented by the item characteristic curve (ICC, [15]). ICC expresses in a nonlinear (logit) regression line how the probability of endorsing an item changes as a function of item difficulty (β), discrimination (*α*) and pseudo-guessing (*c*) parameters. Difficulty (β) indicates the level of the latent trait where there is a 0.5 probability that a participant will endorse a specific criterion or item [17]. For example, ‘easier’ items have lower β values and their ICC is represented closer to the horizontal axis. For clarification purposes, those endorsing easier items are said to have lower BA. Conversely, those who endorse the difficult items are said to have higher BA [15]. Discrimination (*α*) describes how steeply the rate of success (positive response) of an individual varies according to their latent trait levels. Thus, items more strongly related to the latent variable present steeper ICC functions. Finally, pseudo-guessing (*c*) represents the probability of an individual to guess ‘the correct response’ to an item.

**Table 1:** Descriptive statistics for BAS-2 10 items (N = 386)

	Overall				Men	Women
	*M*	*SD*	Skewness	Kurtosis	*M*	*M*
1. I respect my body	3.53	.94	− .32	− .19	3.52	3.55
2. I feel good about my body	3.04	.99	− .13	− .38	3.12	2.95
3. I feel that my body has at least some good qualities	3.60	1.02	− .43	− .30	3.65	3.54
4. I take a positive attitude toward my body	3.21	1.06	− .18	− .54	3.30	3.09
5. I am attentive to my body’s needs	3.41	.95	− .18	− .33	3.44	3.38
6. I feel love for my body	2.93	1.13	.03	− .72	3.05	2.78
7. I appreciate the different and unique characteristics of my body	3.07	1.12	− .07	− .73	3.14	2.98
8. My behaviour reveals my positive attitude toward my body	3.02	1.09	− .01	− .68	3.13	2.89
9. I am comfortable in my body	3.18	1.13	− .23	− .70	3.33	2.99
10. I feel like I am beautiful even if I am different from media images of attractive people	3.05	1.20	− .06	− .85	3.13	2.95

M = mean; SD = Standard Deviation; Min = Minimum; Max = Maximum

^*^ = Statistically significant *p* < .05. Partial invariance achieved by freeing factor loadings 2, 8 and 9, and intercept 2 and 9

While IRT models were originally developed to assess dichotomous data (i.e. yes/no), extensions of these models have been employed to accommodate the use of ordered polytomous data (i.e. more than two response options reflecting order/ranking [15]). Given that the BAS-2 measures BA with a 5-point scale (with multiple and incrementally ordered answers per item), the application of IRT models suitable for polytomous data is required. In that context, “Rasch” models assume equal discrimination (*α*) across items and behave as 1PL models [14]. Alternatively, the generalised partial credit (GPC) and graded response (GR) models assume variable item discrimination properties (*α*), and present more suitable for ordered polytomous data [15, 17]. While the GPC evaluates the probability of responding to one category versus the adjacent category, the GR evaluates the probability that someone grades an item in a higher category score as opposed to a lower category score given their latent trait level (*θ*) [14]. Indeed, non-adjacent models (i.e., GR) have been identified as best fitting models for Likert scales based on their assumption that respondents will choose their best fitting ordered category (e.g. *never* to *always*) in answering an item [17]. Considering that no previous studies have evaluated BAS-2 psychometric properties with either adjacent on non-adjacent polytomous IRT methods, the GR and the GPC models were comparatively employed in the current study.

Finally, considering that the current study attempts to evaluate psychometric properties of the BAS-2 across males and females, an evaluation of differential item functioning (DIF) can be obtained within an IRT framework [15]. Analogous to investigations of MI under a CTT-CFA framework, DIF investigates potential differences in parameters (i.e., *α,* β) across groups of interest (expanding traditional CTT M-CFA MI procedures) [25]. Given that IRT frameworks enable researchers to evaluate item-participant relationships at different levels of the latent trait, DIF facilitates the detection of sources of non-invariance across men and women at high and low levels of BA.

### The present study

Prompted by the revised literature, the present study aims to contribute to the available knowledge related to the psychometric properties of the BAS-2 in two significant ways: (a) it aims to expand gender MI findings via the use of a multi-national sample and the employment of more stringent research methods; and (b) it will be the first to examine the differential item functioning of the BAS-2 items for participants with different levels of BA. Such knowledge is significant in at least three important ways: (a) it will add clarity considering the gender comparability of the BAS-2 scores in both research and clinical practice, while revealing which BA items can be comparatively used for men and women, and which should not be; (b) it will allow ranking of the BAS-2 items based on their psychometric performance (i.e., item priority ranking); and (c) it will inform how the different BAS-2 items may provide reliable and/or less reliable information among participants with higher and lower levels of BA. The latter is deemed of particular importance for populations with clinically low and/or high BA to better inform their treatment based on the BAS-2.

## Methods

### Participants

After receiving ethics approval from the Victoria University Ethics Committee, participants were recruited online, not assuming/following random sampling procedures, via a crowd sourcing platform (Prolific.co) and were awarded $2.50 for their time each. As part of a larger study, 394 participants completed an online survey including the BAS-2. Omission of items was not allowed by the Qualtrics-setting parameters. These included 216 men, 170 women, and 8 participants identified as non-binary. These eight participants were excluded in the present analyses targeting binary gender differences. The remaining participants’ (*N* = 386) age ranged from 18 to 39 years (*M* = 27.54, SD = 5.58). Most participants worked full-time (44.3%), had an undergraduate degree (40.4%), were heterosexual (80.5%), were from the outer metropolitan suburbs (41.7%), reported Caucasian ethnicity (57.8%) and lived in the USA (54.9%).

### Measures

The 10-item BAS-2 [41] uses a 5-point Likert scale with responses ranging from 1 (*Never*) to 5 (*Always*). Higher scores indicate higher BA. To calculate one’s final BA score, item responses are summed, resulting in a score between 5 and 50. Table 1 presents a description of the items and descriptive statistics for the current sample. Previous research found a unidimensional factor structure, along with strong internal consistency (Cronbach’s α = 0.97), construct validity and test–retest reliability (r = 0.90) in community and college samples of men and women [41]. Additionally, the internal consistency of the BAS-2 in the present study was excellent (Cronbach’s α = 0.954, McDonald’s ω = 0.956).

### Statistical analyses

To address the outlined aims, a series of statistical processes were employed: a sequential multigroup CFA to observe MI across men and women; and psychometric examination at the scale and item level via IRT.

First, following previously applied methodology we conducted a multigroup CFA to test for Measurement Invariance (MI) across gender groups (males and females). These analyses were conducted via the Lavaan package in RStudio [29, 30]. This process involves a stepwise model comparison with progressively restrictive parameters to test for ill-fitting models and subsequently observe sources of non-invariance [4]. More specifically, a configural model (factor loadings and intercepts free to vary) is compared with a metric model (factor loadings constrained to be equal across groups and intercepts free), and a scalar model (equal factor loadings and intercepts), respectively. Alongside the stringent nature of *χ*^2^ comparisons between the configural, the metric and the scalar levels (Δ*χ*^2^ < 0.05), we additionally comparatively evaluated incremental fit differences in CFI and RMSEA values (ΔCFI > 0.010, ΔRMSEA > 0.015, [16, 27]). It should be noted that if full measurement invariance is not attained when comparing models, partial invariance can be explored to determine the source of non-invariance by sequentially freeing constrained parameters across different items, until non-significant difference across models is achieved [32].

A combination of statistical processes was applied to determine the source of non-invariance across the different levels. In line with the more stringent approach, the SBSDiff package in RStudio was used to calculate the Satorra-Bentler test of scaled *χ*^2^ difference for factor loadings and intercepts [23]. This test has been identified as appropriate to obtain significant differences between nested and comparison models [31]. Subsequently, modification indices were calculated with RStudio for all contemplated parameters at the metric and scalar levels (factor loadings, *λ*; and item intercept, *α*). Finally, we applied the Benjamini-Hochberg (BH) procedure for controlling false discovery rate in multiple comparisons. The BH procedure has demonstrated superior power of detection when compared with other correction methods (e.g. Bonferroni, Hommel, Hochberg, [37]).

Third, BAS psychometric properties were assessed within the IRT context applying polytomous models. These analyses were conducted utilizing the IRTPRO 5.0 statistical software. IRT models assume that three conditions will be upheld: unidimensionality, local independence, and monotonicity [38]. Firstly, unidimensionality, or appropriate dimensionality, assumes that a single latent trait can appropriately and sufficiently explain the common variance among item responses [15]. Considering that the unidimensional factorial structure of the BAS-2 has been previously evaluated, this study employed CFA to confirm that one latent variable (i.e. in this case BA) appropriately applies. Secondly, local independence assumes that a participant’s response to a question is only conditional to the level of the latent trait, and thus, independent of responses to other items [14]. Chen and Thissen [12] propose that the local dependency (LD) χ^2^ statistic can determine such occurrences by comparing observed and expected frequencies between item responses. Accordingly, LD χ^2^ values larger than 10 could indicate local dependence concerns [12]. This assumption was met as LD χ^2^ BAS-2 items were < 10. Finally, monotonicity refers to the constant increment of a variable as a function of another variable. In IRT contexts, this represents that the probability of endorsing an item should increase as trait levels increase [38]. In other words, a functional form (in this case an ‘S’ shaped curve) should be observed when plotting the function specified by the model [14]. BAS-2 items demonstrated a functional form and thus met the assumption of monotonicity (this can be observed in Fig. 5). Furthermore, IRT models employed included the unidimensional GR and GPC [8]. The GR model deals with ordered polytomous categories and is the preferred method for assessing questionnaires with Likert scales. The GPC estimates partial credit points for correctly endorsing some aspects of the item [26]. Maximum marginal likelihood methods of estimation were employed in line with past recommendations for ordinal polytomous IRT models [7]. Considering the tendency of χ^2^ values to inflate with the use of large sample, as is the case here, the best fitting IRT model was combinedly determined by (i) the loglikelihood index of fit [10], (ii) RMSEA < 0.05 as criteria for sufficient fit [18], and (iii) Bayesian and Akaike Information Criterion (BIC and AIC) with smaller values demonstrating a better model fit [14]. Subsequently, item parameter characteristics were assessed with the Item Characteristic Curve (ICC) and Item Information Function (IIF), while test characteristics were assessed with the Test Information Function (TIF) and the Test Characteristic Curve (TCC [14]).

## Results

### Measurement invariance

**Fig. 1:**
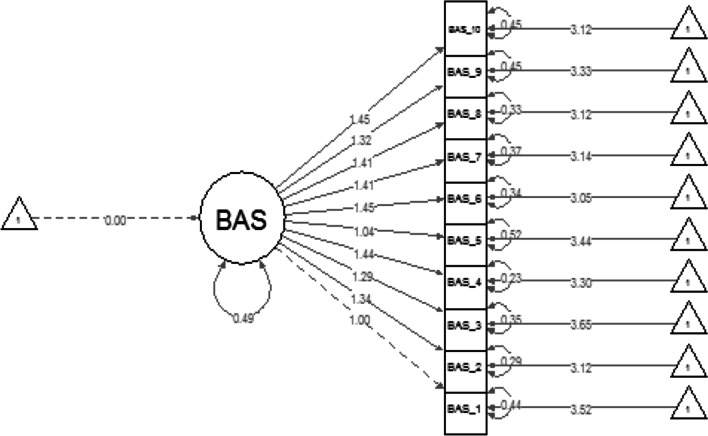
Body appreciation scale unstandardised item loadings for Men. This graph demonstrates the unidimensional factorial structure of the BAS-2 for men

**Fig. 2:**
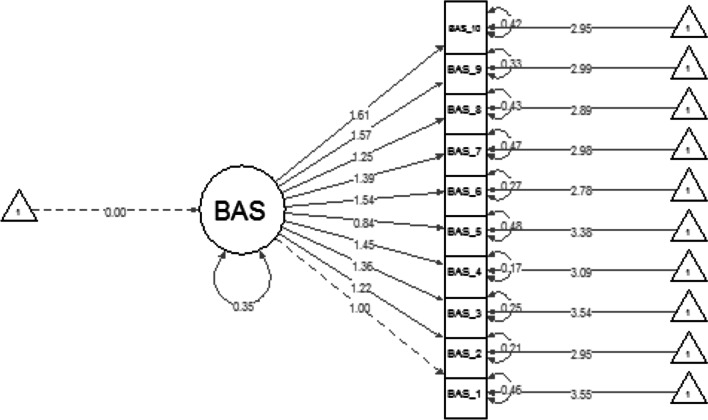
Body appreciation scale unstandardized item loading for Women

First, the BAS unidimensional factorial structure was assessed across gender groups. Both groups demonstrated acceptable fit according to acceptance criteria for RMSEA, TLI and CFI suggested by [18] (males: *χ*^2^ = 80.044, *df* = 35, *p* < 0.001, CFI = 0.972, TLI = 0.963, RMSEA = 0.077; females: *χ*^2^ = 59.404, *df* = 35, *p* = 0.006, CFI = 0.980, TLI = 0.975, RMSEA = 0.064). Unstandardised item loadings for men ranged from 1 to 1.45 (Fig. 1) and for women ranged from 0.84 to 1.61 (Fig. 2). Both groups demonstrated good internal reliability coefficients (males Cronbach’s α = 0.955, and McDonald’s *ω* = 0.957; females α = 0.943, *ω* = 0.946).

Second, MI was conducted for men and women scoring on the BAS. The unidimensional BAS configural model showed acceptable fit for the sample (*χ*^2^ = 161.20, *p* < 0.001, CFI = 0.974, TLI = 0.967, RMSEA = 0.072) with a statistically significant decrease in absolute fit (Satorra–Bentler scaled Δ*χ*^2^ = 19.38, *p* = 0.022) and non-significant change in incremental fit (S-B scaled ΔCFI = 0.004; ΔRMSEA = 0.001) at the metric level (Table 2). Given the significant decrease in absolute fit between configural and metric models, no meaningful observations could be inferred between metric and scalar model comparison. Therefore, we proceeded to identify non-invariant parameters by evaluating modification indices and utilising the Benjamini–Hochberg procedure. As presented in Table 3, parameters that produced a significant S-B scaled Δ*χ*^2^ were λ 2, λ 8, λ 9, α 1 and α 9. After calculating Benjamini–Hochberg adjusted *p* values it was determined that all 5 parameters presented a “*p* < BH *p*” condition, thus remaining significant for partial invariance purposes. Indeed, free estimation of factor loading 2, 8 and 9, and intercepts 1 and 9 achieved a not significant decrease when compared to the configural model (S-B scaled Δ*χ*^2^ = 10.61, *p* = 0.056).

**Table 2:** Test of invariance BAS-2 questionnaire

	*Df*	Δ*Df*	*χ*^2^	Δ*χ*^2^	*p*	CFI	ΔCFI	RMSEA	ΔRMSEA	AIC	BIC
Configural – Model 1 (free loadings, free intercepts)	70		161.20			.974		.072		8249.7	8487.0
Metric – Model 2 (equal loadings, free intercepts)	79	9	177.56	19.38	.022*	.971	.004	.073	.001	8248.0	8449.8
Scalar – Model 3 (equal loadings, equal intercepts)	88	9	193.85	16.1	.063	.968	.003	.072	.001	8246.3	8412.5
Partial Invariance	82	12	172.17	810.61	.056	.975	.001	.071	.001	8236.6	8426.5

All differences (Δ) were calculated obtaining the difference between the current model and the immediate model above except for partial invariance, where the difference was calculated against the configural model

**Table 3:** Benjamini–Hochberg procedure: testing item intercept and factor loadings for BAS invariance between men and women

Model	Parameter Relaxed	*df*	*χ*^2^	*P* value	BH adj *p* value	Sig
*M*_0_		88	193.846			
*M*_*1*_	λ1	86	193.658	.4120	.0112	
*M*_*2*_	λ 2	86	190.588	**.0142**	.0212	*
*M*_*3*_	λ 3	86	193.663	.8657	.0037	
*M*_*4*_	λ 4	86	193.845	.9999	.0012	
*M*_*5*_	λ 5	86	190.325	.0530	.0187	
*M*_*6*_	λ 6	86	192.848	.2388	.0125	
*M*_*7*_	λ 7	86	193.700	.8041	.0062	
*M*_*8*_	λ 8	86	191.137	**.0162**	.0200	*
*M*_*9*_	λ 9	86	186.770	**.0001**	.0025	*
*M*_*10*_	λ 10	86	192.557	.1378	.0162	
*M*_*11*_	α 1	86	187.529	**.0017**	.0237	*
*M*_*12*_	α 2	86	193.821	.1051	.0175	
*M*_*13*_	α 3	86	191.871	.1416	.0150	
*M*_*14*_	α 4	86	193.837	.9769	.0025	
*M*_*15*_	α 5	86	192.915	.4328	.0100	
*M*_*16*_	α 6	86	192.061	.1817	.0137	
*M*_*17*_	α 7	86	193.605	.7888	.0075	
*M*_*18*_	α 8	86	193.189	.5242	.0087	
*M*_*19*_	α 9	86	188.323	.**0040**	.0225	*
*M*_*20*_	α 10	86	193.641	.8427	.0050	

*df* = degrees of freedom; *BH* = *adj p value* Benjamini Hochberg adjusted *p* value. *Sig* = *Significance* is determined by *p* value smaller than BH adj *p* value. Parameter relaxed denotes which parameter has been relaxed in comparison to *M*_*0*_

### Psychometric IRT properties

Following past recommendations [6, 7], we employed marginal likelihood information statistics with one and two-way marginal table to assess goodness of fit (M_2_ [710] = 1443.09, *p* < 0.001, RMSEA = 0.05). Given that M_2_ is sensitive to sample size, RMSEA was sufficient to determine goodness of fit to data [24]. Comparisons across the graded response model (GR) and generalised partial credit model (GPC) were conducted. The GR demonstrated better fit to data (χ^2Loglikelihood^ = 8111.38; RMSEA = 0.06; BIC = 8410.20; AIC = 8211.38) when compared to the GPC (χ^2Loglikelihood^ = 8182.21; RMSEA = 0.06; BIC = 8481.02; AIC = 8282.21), and thus item parameters discussed subsequently were obtained with the GR model. When discrimination parameters (i.e., α) where constrained to be equal across models, a significant decrease in fit indices was observed (χ^2Loglikelihood^ = 11,414.61; BIC = 11,653.66; AIC = 11,494.61). Thus, suggesting that the PC does not appropriately model observed data.

**Fig. 3:**
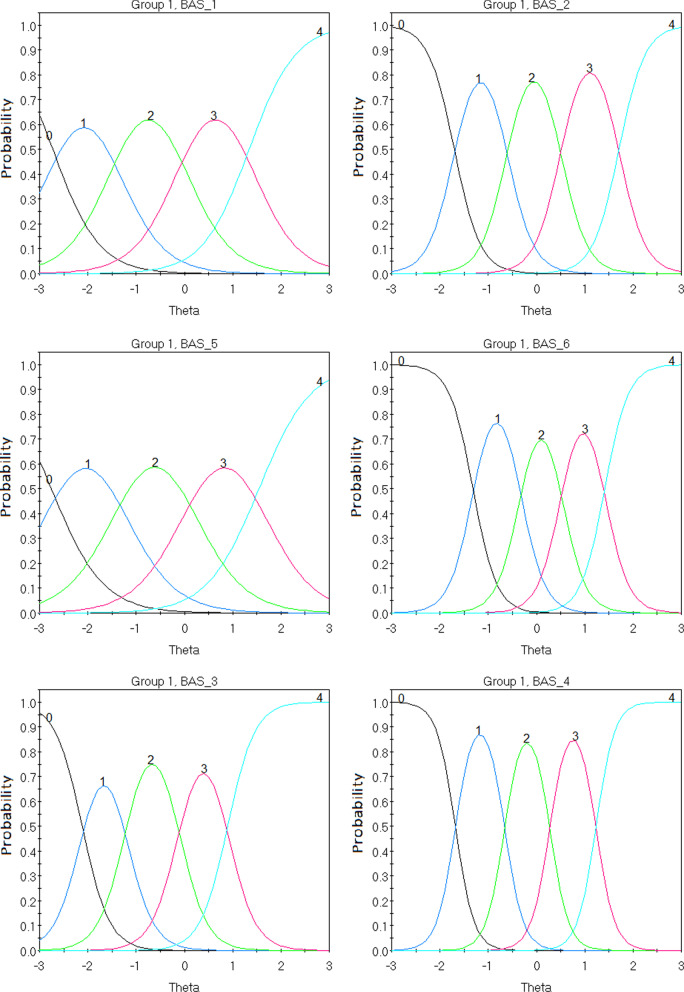

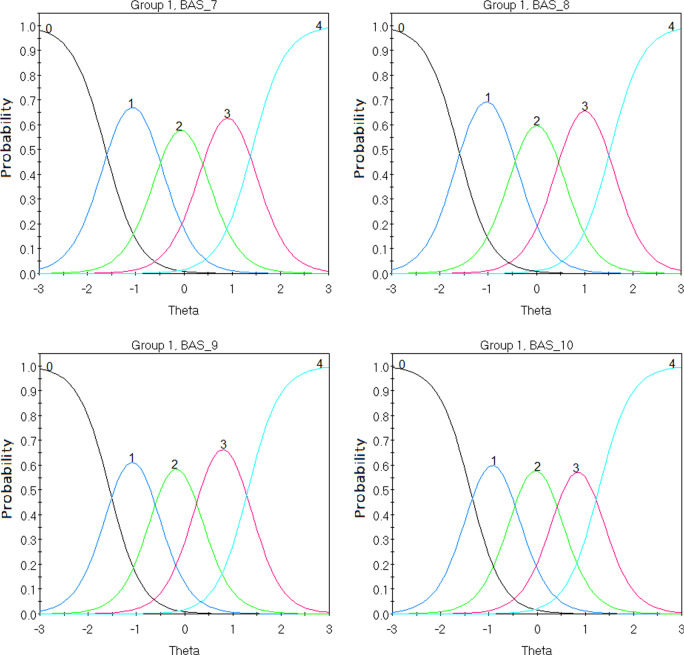
BAS Items’ Characteristic Curves (ICC). These plots demonstrate how the probability of endorsing a category of BAS-2 items (i.e., *never to always*) change as levels of the latent trait change

Discrimination parameters for all ten items fell within the very high range (0 = non discriminative; 0.01–0.34 = very low; 0.35–0.64 = low; 0.65–1.34 = moderate; 1.35–1.69 = high; > 1.70 = very high) between 1.87 (α item 5) and 5.19 (α item 4). The descending sequence of the items’ discrimination power (*α*) is 4, 6, 2, 3, 9, 10, 8, 7, 1 and 5 (see Table 4). Furthermore, the item difficulty parameters (β), demonstrated a considerable level of fluctuations between the different thresholds across the 10 items. Indicatively, for the first threshold the ascending item sequence of difficulty was 6, 10, 9, 8, 2, 4, 3, 1 and 5. Considering the fourth threshold, this alternated to 3, 4, 10, 9, 1, 6, 7, 5, 8 and 2. Nevertheless, the threshold difficulty parameters progressively increased between the first and the last threshold across all items (see Table 4 and Fig. 3). Conclusively, IRT analyses indicated that: (i) while increasing item scores correctly described increasing levels of BA behaviours across all items, the rate of increment is different across the items, and (ii) different thresholds perform differently across items considering their level of difficulty.

**Table 4:** BAS-2 Graded Response Model IRT Properties

Item	*α*	*β*_*1*_	*β*_*2*_	*β*_*3*_	*β*_*4*_
1	2.09	− 2.73	− 1.43	− 0.05	1.34
2	3.71	− 1.70	− 0.61	0.50	1.71
3	3.54	− 2.12	− 1.22	− 0.11	0.89
4	5.19	− 1.68	− 0.66	0.27	1.23
5	1.87	− 2.76	− 1.33	0.11	1.54
6	4.08	− 1.31	− 0.33	0.52	1.41
7	2.90	− 1.63	− 0.51	0.40	1.41
8	2.96	− 1.61	− 0.46	0.48	1.54
9	3.04	− 1.55	− 0.61	0.27	1.32
10	3.01	− 1.38	− 0.46	0.41	1.27

*α* defines the capacity of an item to discriminate between varying levels of body appreciation (θ)

*β* represents the level of latent trait observed to endorse each item at a specific threshold

**Fig. 4:**
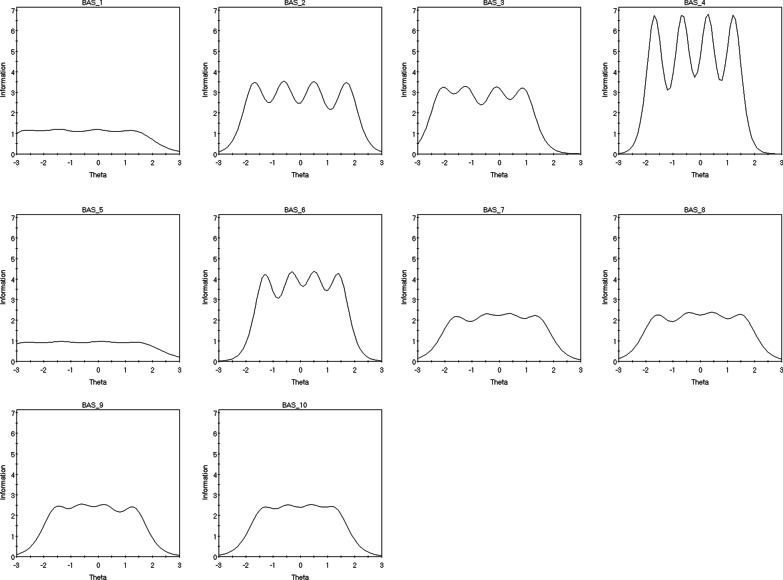
BAS Item Information Function (IIF). These plots demonstrate how reliability indices vary with changes in the latent trait. Interestingly, ‘waves’ of increased information corresponds to item categories

Considering the items’ reliability across the different levels of the latent trait, controlling concurrently for the different levels of items’ difficulty, meaningful variations were confirmed. Indicatively, the IIF of item 4 provided the highest level of information/reliability in the ranges between 2 and 1 and a half SD below and above the mean and the area around half SD below and above the mean. The IIFs of items 2, 6, 9 and 10 showed better performance in the range between 2 SDs above and below the mean (although with some variability of less than 1 point). Items 1 and 5 showed a rather low and undifferentiated level of reliability in the area between minus 3 SDs below the mean and 2 SDs above the mean with significant drop for behaviours exceeding 2 SDs above the mean. Finally, item 7 showed average reliability for the area between 2 SDs above and below the mean and significant drop for score around 3 SDs above or below the mean (see Fig. 4).

**Fig. 5:**
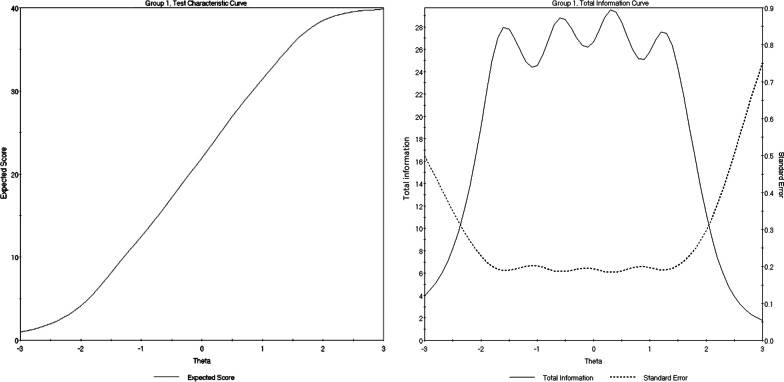
BAS Test Characteristic Curve (TCC) and Test Information Function (TIF). The TCC provides a visual representation of expected BAS-2 scores as a function of latent trait levels (i.e., as BAS-2 scores increase, levels of the latent trait increase). The TIF demonstrates the relationship between standard errors and reliability indices (i.e., smaller standard errors result in more information)

The performance of the scale as whole is visualized by the Test Characteristic Curve (TCC) and the Test Information Function (TIF). The TCC graph illustrates that the trait of BA inclined steeply, as the total score reported increased (from 4 to 49; see Fig. 5). Considering the information provided by the scale, improved information (TIF) scores were around 2 SDs below the mean, up to about 2 SDs above the mean (see Fig. 5).

These results suggest that the scale (as a whole) provides a sufficient and reliable psychometric measure for assessing individuals with high and low levels of the BA behaviours in the range between 2 SDs below and above the mean. Nevertheless, it may not be an ideal measure for extremely low and high BA in the areas around 3 SDs above and below the mean. The BA behaviour at the levels of 2 SDs below and above the mean trait level correspond with raw scores of 4 and 39 respectively, and based on these, they could be suggested as conditional (before clinical assessment confirmation) diagnostic cut-off points.

**Fig. 6:**
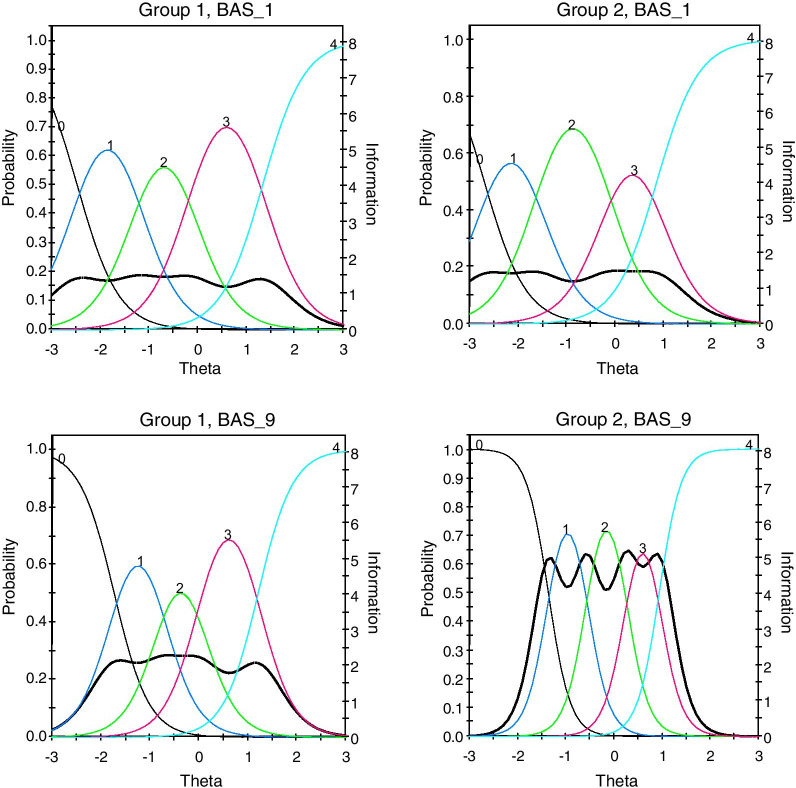
DIF for non-invariant items across men (group 1) and women (group 2). Continuous lines represent different categories of responses (i.e., *never to always*). Dotted lines represent reliability indices

Considering DIF of BAS-2 across men and women, sources of non-invariance at the item level were detected. DIF statistics were observed (Table 5) for all items with significant discrepancies (*p* = 0.05) in items 1 and 9 across men and women including all parameters (χ^2^), items 3 and 9 including only discrimination (χ^2^_a_), and item 1 including only difficulty (χ^2^_cja_). Following past recommendations [25], we anchored invariant items and calculated DIF statistics with only non-invariant items to avoid increasing type I error. As observed in Table 5, non-invariance was observed for both items (1 and 9) including all parameters, with a significant difference including only discrimination (*p* = 0.01) in item 9, and a significant difference including only difficulty (*p* = 0.01) in item 1. That is, endorsing categories in item 1 requires lower levels of BA in women. Figure 6 offers a visual representation of this relationship. For example, in item 1 men with 1 SD below the mean are more likely to endorse category 1 (*seldom*) than women with 1 SD below the mean. Similarly, women with 1 SD above the mean are more likely to endorse category 4 (*always*) compared to men. In item 9 however, significantly different discrimination (*α*) indicates that women with 2 SD below the mean are more likely to endorse category 0 (*never*) compared to men, and this relationship is reversed as levels of BA increase (i.e., women with 2 SD above the mean are more likely to endorse category 4 [*always*] compared to men).

**Table 5:** DIF statistics across men and women

Item number	Total χ^2^	*df*	*p*	χ^2^_a_	*df*	*p*	χ^2^_cja_	*df*	*p*
1	14.2	5	**0.0142**	0.0	1	0.8646	14.2	4	0.0067
2	5.8	5	0.3257	0.9	1	0.3382	4.9	4	0.2992
3	8.0	5	0.1542	5.1	1	0.0238	2.9	4	0.5711
4	3.5	5	0.6186	2.3	1	0.1316	1.3	4	0.8697
5	4.1	5	0.5407	1.2	1	0.2771	2.9	4	0.5785
6	2.9	5	0.7237	1.4	1	0.2439	1.5	4	0.8292
7	2.2	5	0.8201	0.3	1	0.5763	1.9	4	0.7555
8	3.6	5	0.6019	2.1	1	0.1491	1.6	4	0.8167
9	11.7	5	**0.0385**	5.4	1	0.0201	6.3	4	0.1747
10	2.1	5	0.8314	1.3	1	0.2496	0.8	4	0.9387

Item number	Total χ^2^	*df*	*p*	χ^2^_a_	*df*	*p*	χ^2^_cja_	*df*	*p*
*DIF statistics including only non-anchored items*									
1	13.2	5	0.0214	0.0	1	0.9721	13.2	4	0.0102
9	12.5	5	0.0285	6.0	1	0.0142	6.5	4	0.1656

Item number	Group	*α*	β_1_	β_2_	β_3_	β_4_			
*BAS-2 Graded Response Model IRT Properties for non-invariant items*									
1	Men	2.34	− 2.47	− 1.23	− 0.14	1.33			
1	Women	2.33	− 2.69	− 1.59	− 0.14	0.86			
9	Men	2.84	− 1.72	− 0.76	0.02	1.21			
9	Women	4.41	− 1.36	− 0.57	0.25	0.93			

Bold values denote significance at .05 and are considered ‘non-anchored items’ in the table immediately below

α defines the capacity of an item to discriminate between varying levels of body appreciation (θ).

β represents the level of latent trait observed to endorse each item at a specific threshold.

## Discussion

The present study is the first of this type to combine CTT and IRT procedures to assess BAS-2 psychometric properties at both the scale and the item level for an English-speaking sample. Considering MI, the loadings of items 2, 8 and 9, and the intercepts of items 1 and 9 were shown to be non-invariant across males and females, when strict (*χ*^2^) comparisons were applied. Considering the IRT evaluation, and although all items presented with high discrimination capacity, this fluctuated according to the following descending sequence of items 4, 6, 2, 3, 9, 10, 8, 7, 1 and 5. Similarly, items’ difficulty parameters differed across the different item thresholds. Finally, although the scale as a whole seems to perform sufficiently and reliably when examining BA levels that lie ∓ 2 SD beyond the mean, it is not ideal for extremely low and high levels of BA that lie ∓ 3 SD beyond the mean.

### Uni-dimensionality and measurement invariance across genders

In line with past studies, BAS-2 demonstrated an appropriate unidimensional factorial structure with all items loading saliently and significantly on a single latent variable [20, 28, 36, 41]. When dividing the sample into men and women, BAS-2 maintained an appropriate unidimensional factorial structure with all items loading significantly and acceptable model fit indices for both groups. Further, when using a ‘relaxed’ approach (i.e., changes in CFI and RMSEA, [4]) to establish invariance across gender groups, BAS-2 demonstrated support for invariance at configural, metric and scalar levels. However, when contemplating a more ‘stringent’ approach (S-B Scaled Δ*χ*^2^), non-invariance at the metric and scalar levels was observed. Lack of full MI has been similarly observed in a USA sample [40]. Thus, although BA is perceived in the same unidimensional way across binary genders, cautious comparisons need to be attempted due to different gender response patterns across the different items.

Specifically, support for partial invariance revealed that the degree of relationship between BA and items 1, 3, 4, 5, 6, 7 and 10 is equivalent for males and females. Nevertheless, the BA relationship with items 2 (I feel good about my body), 8 (My behaviour reveals my positive attitude toward my body) and 9 (I am comfortable in my body) is unequally associated with males and females due to different response styles. Thus, the metric utilised for BA measurement is non-equivalent across the two gender groups and thus comparisons based on the responses of these items need to be avoided, or carefully interpreted.

Further, the observed support for partial invariance suggested that sources of non-invariance across gender groups were also present in item intercepts. While items 2, 3, 4, 5, 6, 7 and 8 were invariant, items 1 (*“I respect my body”*) and 9 (*“I am comfortable in my body”*). demonstrated unequal intercepts between men and women. That is, while women are expected to score higher ratings of item 1 (*“I respect my body”*) at all levels of the latent variable, men are expected to score higher ratings of item 9 *(“I am comfortable in my body”*). Interestingly, Tylka and colleagues [41] found a similar source of non-invariance for item 1 in a USA sample. This suggests that males and females who experience the same level of BA may provide unequal responses for this particular item (i.e., gender specific item scaling).

In addition, DIF statistics employing an IRT framework confirmed that items 1 and 9 do not measure BA in the same manner for men and women. Given that women are expected to score higher ratings of item 1, lower levels of BA are required to endorse each category (i.e., *never, seldom, sometimes,* etc*.*). Moreover, significantly different discrimination parameters between men and women in item 9 suggest that at similar levels of the latent trait men and women respond differently. That is, women with low levels of BA are more likely to endorse not feeling *“comfortable in [her] body”* compared to men. Similarly, women with high levels of BA are more likely to endorse feeling *“comfortable in [her] body”* compared to men. These results further suggest that item 1 and 9 should be calibrated/revised to obtain comparable levels of BA across men and women with the BAS-2.

### Scale and item discrimination, difficulty, and reliability

IRT findings identified variability across BAS-2 items when considering different levels of BA within participants. Considering that IRT principles relate to the identification of most appropriate items for the evaluation of a specific level of a latent trait, items were evaluated and ranked in relation to their discrimination, difficulty, and reliability [15]. The descending order of items’ discrimination power was 4, 6, 2, 3, 9, 10, 8, 7, 1 and 5, suggesting that items invoking positive feelings (item 6 “*I feel love for my body*”, item 2 “*I feel good about my body*”, and item 3 “*I feel that my body* …”) and clear statements reflecting dispositional attitude (item 4 “*I take a positive attitude toward my body*”) are able to capture BA levels more effectively as the criterion increases in the individual. Further, while the level of difficulty of endorsing an item increased between the first (*never*) and last options (*always*) of the Likert scale, the sequence of item difficulty varied across thresholds. That is, the ascending order of endorsed items between the first (*never*) and second (*seldom*) options of the Likert scale was 6, 10, 9, 8, 2, 4, 3, 1, and 5. However, the ascending order of endorsed items between the fourth (“*often”*) and last (“*always”*) options of the Likert scale was 3, 4, 10, 9, 1, 6, 7, 5, 8, and 2. This suggests that participants felt more inclined to endorse “*never”* or “*seldom”* loving their body or feeling beautiful than respecting their body or being attentive to their body needs. Alternatively, participants felt more inclined to endorse *“often*” or *“always*” seeing good qualities and taking a positive attitude towards their bodies than feeling good about their bodies. Therefore, it is proposed that items should be interpreted differently when conducting clinical assessment of BA.

Considering the scale (TIF), improved information performance was observed in the range between 2 SDs below and above the mean. However, considerable variation was observed in relation to the level of information precision provided by each criterion. More specifically, findings demonstrated that item 4 (*“I take a positive attitude toward my body”*) provided the highest level of information/reliability between 2 SD below and 1.5 SD above the mean. Items 2 (*“I feel good about my body”*), 6 (*“I feel love for my body”*)*,* 9 (*“I am comfortable in my body”*) and 10 (*“I feel like I am beautiful even if I am different from media images of attractive people”*) provided a considerable amount of information/reliability between 2 SDs below and above the mean. Finally, items 1(*“I respect my body”*) and 5 (*“I am attentive to my body’s needs”*) provided a consistently low amount of information/reliability between 3 SDs below and above the mean. However, these two items along with item 3 (*“I feel that my body has at least some good qualities”*) provided the most information between 2 and 3 SDs below the mean. This indicates that the following three-item sequence should be prioritised when attempting to identify participants with significantly low BA: (i) “*I feel that my body has at least some good qualities*”, (ii) “*I respect my body*”, and (iii) “*I am attentive to my body needs*”. Lastly, the Test Characteristic Curve (TCC) demonstrated an appropriate steepness indicating that BAS-2 clearly identifies increments in BA as the overall score increases. This favours BAS-2 as a sufficient psychometric measure for the assessment of individuals with high and low levels of BA. Nonetheless, the instruments performance significantly decreases to differentiate very low (-3 SD) and very high (+ 3 SD) BA levels.

## Conclusion, limitations and further research

Overall, the present findings suggest that BA comparisons across gender based on BAS-2 should be cautiously interpreted due to response pattern differences affecting the metric and the scale properties of the instrument. Furthermore, the instrument may not perform well for clinically low and high BA levels and thus its use should be accompanied by clinical interviews for formal assessment. While scores for individuals on both ends of the spectrum cannot be discarded, caution must be applied. Therefore, complementing this assessment with alternative questionnaires might provide clarification for such extreme scores. Last, items differ considering their suitability to discriminate participants with different levels of the latent trait with certain items.

Despite the unique and innovative contribution this study makes to the evaluation of BAS-2 psychometric properties, a number of limitations should be highlighted. The employed sample encompassed adult English speakers from developed countries. That is, findings observed in the current study might lack a wide generalisability of application to samples involving youth, and non-English speakers. In addition, considering that a community sample of healthy adults was employed, reported IRT properties might not accurately reflect those suffering from pathological body dissatisfaction. Future studies may wish to address these limitations to improve assessment procedures informed by BAS-2. In that line, it might also be useful for the clinical application of the scale, to additionally estimate a single index of precision/reliability applicable to BAS-2 scores regardless of one’s level of BA, such as marginal reliability and empirical reliability [5].

## Data Availability

Dataset can be made available upon reasonable request.
